# Propolis Consumption Reduces *Nosema ceranae* Infection of European Honey Bees (*Apis mellifera*)

**DOI:** 10.3390/insects11020124

**Published:** 2020-02-15

**Authors:** Alessandra Mura, Michelina Pusceddu, Panagiotis Theodorou, Alberto Angioni, Ignazio Floris, Robert J. Paxton, Alberto Satta

**Affiliations:** 1 Department of Agricultural Sciences, University of Sassari, 07100 Sassari, Italy; amura1@uniss.it (A.M.); mpusceddu@uniss.it (M.P.); ifloris@uniss.it (I.F.); 2 General Zoology, Institute of Biology, Martin Luther University Halle-Wittenberg, Hoher Weg 8, 06120 Halle (Saale), Germany; panatheod@gmail.com (P.T.); robert.paxton@zoologie.uni-halle.de (R.J.P.); 3 Department of Life and Environmental Sciences, University of Cagliari, 09124 Cagliari, Italy; aangioni@unica.it

**Keywords:** ethanol extract, honey bee health, infection, microsporidia, nosemosis, self-medication

## Abstract

*Nosema ceranae* is a widespread obligate intracellular parasite of the ventriculus of many species of honey bee (*Apis*), including the Western honey bee *Apis mellifera*, in which it may lead to colony death. It can be controlled in *A. mellifera* by feeding the antibiotic fumagillin to a colony, though this product is toxic to humans and its use has now been banned in many countries, so in beekeeping, there exists a need for alternative and safe products effective against *N. ceranae*. Honeybees produce propolis from resinous substances collected from plants and use it to protect their nest from parasites and pathogens; propolis is thought to decrease the microbial load of the hive. We hypothesized that propolis might also reduce *N. ceranae* infection of individual bees and that they might consume propolis as a form of self-medication. To test these hypotheses, we evaluated the effects of an ethanolic extract of propolis administered orally on the longevity and spore load of experimentally *N. ceranae*-infected worker bees and also tested whether infected bees were more attracted to, and consumed a greater proportion of, a diet containing propolis in comparison to uninfected bees. Propolis extracts and ethanol (solvent control) increased the lifespan of *N. ceranae*-infected bees, but only propolis extract significantly reduced spore load. Our propolis extract primarily contained derivatives of caffeic acid, ferulic acid, ellagic acid and quercetin. Choice, scan sampling and food consumption tests did not reveal any preference of *N. ceranae*-infected bees for commercial candy containing propolis. Our research supports the hypothesis that propolis represents an effective and safe product to control *N. ceranae* but worker bees seem not to use it to self-medicate when infected with this pathogen.

## 1. Introduction

The microsporidian *Nosema ceranae*, first isolated in *Apis cerana* [1], is an obligate gut parasite of several *Apis* (true honey bee) species [2]. It was first identified as an infective agent of *Apis mellifera* in Spain [3], but the analysis of preserved specimens of *A. mellifera* suggest its presence in Europe as early as 1993 [4]. It nowadays has a worldwide distribution [5,6]. In Italy, this parasite seems to have completely replaced the congeneric parasite *Nosema apis*, which had historically been the only *Nosema* species present [7], possibly through its competitive superiority in warmer climates [8].

*N. ceranae* infection can cause many physiological and behavioural changes at the individual honey bee level [9,10,11,12]. It can also cause pathology at the colony level [13] and it has been associated with colony collapse [14,15,16]. *N. ceranae*, as other microsporidia, is transmitted horizontally via the faecal-oral route, and an infection can occur by the ingestion of spores in food reserves and via trophallaxis between nestmates within the colony [17]. In addition, Sulborska et al. [18] have recently demonstrated that the spores of this pathogen can be carried in the air and deposited on various surfaces in the natural environment, including flowers, thereby further increasing the risk of infection for individual bees and colonies. For beekeeping, effective control treatments against *Nosema* infections are needed. In the last decades, fumagillin has been used to treat colonies infected by *N. apis* [19]. However, several studies have suggested that this antibiotic may be ineffective against *N. ceranae* [20,21,22]. In addition, the toxicity of fumagillin to humans represents another restriction in its application in beekeeping [19]. These concerns led the European Union to ban the use of fumagillin in agriculture in 2010 [23]. This heightens demand for new and safe products that are effective against *N. ceranae*. Though many substances have been assayed in laboratory or field conditions for their efficacy in reducing *N. ceranae* infections, with encouraging results [24,25,26], further studies are needed to develop alternatives to combat *N. ceranae* infections [27].

Among the various promising substances to control *N. ceranae* infection, orally administered organic extracts and natural supplements deserve particular attention due to their putative low toxicity and beneficial effects in increasing bee longevity and in decreasing *Nosema* spore loads [24,26,28,29,30]. Honey bees also forage on many compounds that can have a positive impact on colony health [31]. Among them, propolis is a resinous mixture of substances with antimicrobial properties that is collected from plants by several Hymenoptera species and used by them to protect their nests from parasites and pathogens [32,33,34,35]. As recently suggested [36], honey bees may also obtain propolis directly during digestion of pollen [37].

In feral honey bee colonies, a thin layer of propolis covers the entire nest’s internal walls, whereas in commercial hives a more irregular distribution of propolis is observed. This is possibly because the smoothness of the inner walls of commercial hives does not elicit propolis deposition behaviour. In both feral and managed colonies, propolis is also used for covering holes and crevices in the nest and to limit access to the hive [32,35,38]. In commercial hives enriched with propolis, their microbial load was reduced, resulting in a significant down-regulation of immune function [39]. This decreased energetic investment in the bee immune system can positively influence colony health and productivity [40,41,42,43]. Moreover, propolis can have a direct effect against some hive pathogens, such as *Paenibacillus larvae*, the causative agent of the American foulbrood [44,45], and *Ascosphaera apis*, the causative agent of chalkbrood infection [46].

Interestingly, an increase in resin collection was observed in honey bees after infection with the fungus *Ascosphaera apis* [46] and after an increase in colony infestation by *Varroa destructor* [47]. Natural propolis also reduced DWV viral loads associated with *V. destructor* infestations at the colony level [48]. However, increased resin collection does not appear to be associated with infection by the American foulbrood agent *Paenibacillus larvae* [46]. Honeybee nurse bees infected with the microsporidian gut parasite *N. ceranae* preferred honeys with a higher antibiotic activity, which reduced microsporidian infection after consumption of the honey [49]. Although it is not known whether honey bees consume propolis, Turcatto et al. [50] demonstrated that adding propolis to the diet of bees injected with *Escherichia coli* caused significant up-regulation in antimicrobial gene expression (defensin-1, abaecin, hymenoptaecin, and apidaecin) compared to that observed in infected bees fed a similar diet without propolis. This increase did not occur in uninfected bees fed propolis, suggesting that propolis may enhance a bee’s response when challenged by pathogens. These findings suggest that therapeutic use of propolis in the hive may control *N. ceranae*. On the other hand, propolis can have negative effects on honeybees; when orally administered in sucrose syrup at a concentration of 20%, it caused 25% bee mortality after 72 h [51] and at a concentration of 10% it caused sub-lethal effects, such as alteration of protein dynamics in the fat bodies and depletion of the hypopharyngeal glands [52]. In addition, bees fed propolis in sucrose syrup showed elevated expression of three CYP6AS cytochrome P450 genes involved in pesticide detoxification [53], suggesting that propolis may have a mildly toxic effect on individual bees. We, therefore, hypothesize that bees should consume moderate amounts of propolis only when stressed, such as when challenged by a pathogen.

Recently, the therapeutic properties of propolis extracts against *N. ceranae* were assayed in the Asiatic honey bee species *Apis florea* [54,55], *Apis cerana* [56] and the Western *Apis mellifera* [57]. In the Asiatic *Apis* species, propolis used in the bioassays was obtained from the stingless bee *Trigona apicalis*, whereas in *A. mellifera* the propolis tested was produced by honey bees themselves. In all these studies, an improvement in bee survival and a decrease in the *N. ceranae* spore load were observed in experimentally infected bees [54,55,56,57]. For the Asiatic *Apis* species, these results leave open the question of whether the honey bee’s own propolis has a similar beneficial effect in reducing *Nosema* pathogenesis. For *A. mellifera*, an open question remains as to whether propolis *per se* or the solvent used to dissolve and administer the propolis, or both, caused an improvement in bee health.

To resolve these open questions, we here tested the effects of propolis produced by honey bees (*A. mellifera*) in Sardinia (Italy), an island with a typical Mediterranean climate, on the longevity of *A. mellifera* experimentally infected with *N. ceranae* and on their microsporidian spore load. Secondly, we ascertained whether infected bees were more attracted to a diet containing propolis and consumed a greater proportion of this diet in comparison to uninfected bees. Considering that the biological properties of propolis have mainly been associated with its phenolic components [43,58,59,60], we also quantified the polyphenol content together with the main phenolic compounds of the propolis used in our bioassays.

## 2. Materials and Methods 

### 2.1. Sources of Honey Bees and Propolis 

Honey bees and propolis used in this study were collected from the hives of the experimental apiary of the Dipartimento di Agraria of the University of Sassari, located in Ottava, Sardinia, Italy (latitude 40°46′23″ N, longitude 8°29′34″ E). The flora of the area, described in detail by Bagella and Urbani [61] and Biondi et al. [62], is mainly composed of species typical of the thermophilic Mediterranean maquis at various stages of succession (*Pistacio-Querceto ilicis* plant community characterized by the presence of *Chamaerops humilis*) in a patchwork pattern with traditional agricultural land uses (olive groves, vineyards and cereal crops). The apiary consisted of 15 colonies of *Apis mellifera ligustica* Spinola maintained in Dadant hives each containing 10 combs. During the experimental period (from May to November 2018), colonies were checked regularly to verify the presence of the queen and supplies of pollen and nectar, and to check the health of the bees and that they were devoid of *Nosema* following methods in Fries et al. [63]. Propolis samples used in the bioassays were collected from twelve hives from May to July 2018 by using fine nylon mesh placed above the combs. After scraping propolis from the mesh, each sample was cleaned of impurities, weighed and stored in a freezer at −18 °C. Before starting bioassays, all frozen samples of propolis were homogenized using a coffee mill (GS Arendo, Hannover, Germany).

### 2.2. Propolis Extract Preparation

To obtain the propolis extract, 2.4 g of crude propolis were dissolved in 4 mL of 70% ethanol and the solution maintained in a chamber at 31 °C in the dark for 24 h [54]. Then, the propolis extract was filtered by suction to clean it from wax and other impurities [64]. Based on previous studies [54,56], a concentration of 50% of this extract in distilled water (v/v) was prepared for the experiments.

### 2.3. Pathogen Preparation

*Nosema ceranae* spores used in bioassays were first propagated in the laboratory through mass feeding of caged honey bees with *N. ceranae* spores originating from infected bees provided by the Institute CREA—Consiglio per la Ricerca in Agricoltura e l’Analisi dell’Economia agraria—Unità di Ricerca in Apicoltura e Bachicoltura (Bologna, Italy). Inocula were prepared freshly on the day of experimental bee infection by crushing the ventriculus of infected honey bees in distilled water and purifying using Percoll^®^ following standard procedures [63]. Spore numbers were counted with a Neubauer haemocytometer (Brand®, Werthein, Germany) under a light microscope (×400) and diluted to obtain the required concentration (10^5^ per µL) in 50% (w/v) sucrose solution. For the control treatment, an extract from the ventriculus of uninfected caged honey bees was obtained as above. Before starting the propagation, the identification *N. ceranae* in the inoculum was confirmed by PCR [63] and the absence of spores in control bees was checked by inspection of their ventriculi under a light microscope (×400).

### 2.4. Survival Bioassay Set-Up

To obtain adult workers and perform the laboratory bioassays, frames of honey bee brood ready to emerge were collected from three *N. ceranae*-free colonies and kept for 14 h in an incubator at 35 °C and 70% relative humidity (RH). Each freshly emerged bee was individually fed with 2 µl of one of the following six treatments obtained by mixing at a 1:1 ratio a 50% (w/v) sucrose solution with: (1) aqueous homogenate of healthy bee gut homogenate (Control); (2) 35% ethanol solvent (Control + Ethanol); (3) 50% propolis extract in ethanol (Control + Propolis); (4) gut homogenate with 10^5^ *N. ceranae* spores per bee (Nosema), (5) 35% ethanol solution with 10^5^ *N. ceranae* spores per bee (Nosema + Ethanol), and (6) 50% propolis extract with 10^5^ *N. ceranae* spores per bee (Nosema + Propolis). Once the spores were purified, the time required for the preparation of the treatments and their administration to the bees was approximately 4 h.

After feeding, each bee was placed inside a perforated microfuge tube for 30 min to check if the inoculum had been eaten. Bees that did not eat all the food supplied, had regurgitated inoculum or did not appear healthy were eliminated from the bioassay. The number of spores used to infect bees (10^5^ per bee) was chosen to ensure infection of every individual [65,66]. We performed the bioassay using three replicates of 21 bees each (seven bees from three colonies, equally mixed in a cage to eliminate colony effects) per treatment. Each group was placed in a metal cage (10 cm × 10 cm × 5 cm) with perforated walls. Bees were kept in an incubator at 31 °C and fed ad libitum with 50% (w/v) sucrose solution administered using a syringe (Sterile Siring PIC, 5 mL, Pic Solution, Como, Italy) [67]. Dead bees were counted daily and removed from cages. All experimental treatments were performed at the same time. The bioassay ended on the 30th day of observation. 

### 2.5. Food Choice Test

Adult bees used in the choice test were obtained as described in the survival experiment. Freshly emerged bees were split into two groups of 60 individuals each. One group (infected group) was mass fed with 2 mL of a 50% (w/v) sucrose solution containing 10^5^ *N. ceranae* spores per bee, sufficient to guarantee 100% infection. When the food containing the spores was completely consumed, the bees were fed with sucrose solution (50% w/v) administered ad libitum until the start of observations, which was chosen to be 4 days after spore administration in order to be sure that the infection of the epithelial cells had already started [68]. The control group received only sucrose solution (50% w/v) administered *ad libitum* until the beginning of observations.

The attractiveness of a commercial protein candy (Chemicals LAIF s.p.a, Vigonza (PD), Italy) and to which we had added coffee-milled crude propolis was compared with that of the same candy without propolis in infected and healthy bees. The candy was placed on squares (4 × 4 cm) of filter paper of 67 g/m^2^ (APTACA SRL, Canelli, Italy) in a Y-shaped olfactometer with a transparent plexiglass cover (main arm: 25 cm length, 20 cm width, 10 cm height; each side-arm: 20 cm length, 20 cm width, 10 cm height). Two separate experiments were conducted under artificial light at 27 ± 1 °C to compare the candy with crude propolis at two different concentrations (2% in one experiment and 5% in the other) versus the candy without propolis. The two concentrations chosen were below those known to cause lethal and sublethal effects on adult bees [51,52]. In each test, candy devoid of propolis was given in one arm and candy with propolis (at 2% or 5%) was offered in the other arm. Starting from the fourth day after infection, bees from the *Nosema*-infected or control groups were tested across the subsequent three days. Each bee was placed carefully inside the main arm through a circular hole (1.5 cm diameter) using soft tweezers and then allowed to walk freely in all three arms. Every trial was terminated when a bee arrived at one of the two arm ends, containing either control or propolis-laced candy, and extended its proboscis. A total of 60 control bees and 60 infected bees were tested for each experiment (2% or 5% propolis concentration). Each bee was tested only once and those that did not choose either of the two candies within 10 min (“no choice” subjects) were excluded from statistical analysis. Infected and control bees were tested alternately and, for each bee belonging to the same treatment group, we exchanged the position of the two types of candy.

### 2.6. Scan Bees Sampling and Food Consumption Test

*Nosema* infected and control bees were obtained following the same procedure described in the Food Choice Test (see above). Four days after spore administration, we placed 10 to 15 bees belonging to the *Nosema*-infected or control groups in a metal cage containing 1 g of two different candies (one with and one without propolis). After each hour for 8 consecutive hours, the number of bees eating the two types of candy (control and either 2% propolis or 5% propolis candy) was recorded. To estimate the total food consumed, each candy was weighed at the beginning and the end of the bioassay. The proportion of bees feeding on the two types of candy and the relative amount of propolis consumed (calculated as the propolis-candy consumed/total candy consumed) were compared between the infected and the control groups. The bioassays were conducted from the 4th day post infection (dpi) and finished after three consecutive days (i.e., 7 dpi). We carried out bioassays to test candy at two crude propolis concentrations (2% and 5%) and each treatment was replicated using 3 independent cages.

### 2.7. Quantifying N. ceranae Infection: DNA Extraction and qPCR

In the survival bioassay, we sampled one bee per treatment from each cage after 7 and 14 days post infection (36 bees in total) to quantify the intensity of *N. ceranae* infection (as genome equivalents). The intensity of infection was determined using whole bee DNA extracts. Bees were individually crushed in 500 µL DEPC-H_2_O, and then 100 µL were used for genomic DNA extraction using a DNeasy^®^ Plant Mini Kit (Qiagen) according to the manufacturer’s instructions. To quantify *N. ceranae* infection, we used the following qPCR primers, previously described by vanEngelsdorp et al. [69] and with a modification to the reverse primer by [8]: Forward (5’-3’): CAATATTTTATTATTTTGAGAGA; Reverse (5’-3’): TATATCTATTGTATTGCGCGTGCA giving an amplicon length of 232 bp for *Nosema ceranae*. PCR reactions were performed in a Bio-Rad C1000 Thermal Cycler (Bio-Rad, Hercules, CA, USA) using 2× SensiMixTM SYBR and Fluorescein (Bioline, Memphis, TN, USA), 0.2 µM of each primer and 1 µL of template in a final volume of 10 µL. A negative control without template was included in each run. Each reaction was performed in duplicate and the average quantification cycle (*Cq*) value was taken (accepting a maximum *Cq* difference of 1 between duplicates). Amplification was performed using the thermal profile described in van Engelsdorp et al. [69] with an optimal annealing temperature of 54 °C. Post amplification melt curve analysis was used to check for non-specific amplification (50 °C to 95 °C with an increase of 0.5 °C per second). Standard curves were included in each run for absolute quantification of DNA copy number (genome equivalents) of *N. ceranae* using the methods and DNA dilution series described previously [8]; we accepted PCR efficiencies between 90% and 100%.

In the choice and scan sampling bioassays of food consumption, we ascertained the presence of *N. ceranae* in infected groups and its absence in control groups by sampling one bee per cage for the control groups on days 4 and 7 post treatment and two bees per cage on days 4 and 7 from each propolis treatment (2% and 5%). DNA was extracted from individual bees and a standard PCR for presence/absence was performed using the same protocol as described above for qPCR [63]. PCR products were visualised on 1.5% agarose gels after staining with EZ-Vision two^®^ (Amresco, Radnor, PA, USA).

### 2.8. Chemical Analyses of Propolis 

To determine the chemical composition of our propolis, an HPLC 1100 system equipped with a DAD detector G1315A, an autosampler G1313A, a pump G1311A, and a column oven G1316 (Agilent Technologies, Milan, Italy) were used. The system was controlled by the HP CHEMSTATION for LC software. The wavelengths monitored were 280, 360, and 520 nm. The column was a Varian Polaris C18 (5 µm, 300 A, 250 mm × 4.6 mm). The solvents used were 0.22 M phosphoric acid (A) and acetonitrile/methanol (1/1, v/v) (B). The gradient used for separation and analysis was the following: T = 0 A 96% A; T = 40 50% A; T = 45 40% A; T = 60 0% A, hold for 10 min; at the end of the analysis the column was reconditioned at the initial conditions for 15 min. The flow was 1 mL/min. Identification of the compounds was made using certified analytical standards supplied by Sigma Aldrich (St. Louis, MO, USA). Quantification was made by plotting area versus concentration of compounds in the sample versus five-point calibration curves made with authentic standards.

The total polyphenol content of propolis was determined by the Folin-Ciocalteu method [70]. In brief, solutions for analysis were prepared by reacting 100 μL of the propolis solution or standard with 500 μL of Folin-Ciocalteau reagent for 5 min and then adding 3 mL of 10% (w/v) sodium carbonate solution and ultrapure water up to a final volume of 10 mL. After 90 min incubation at room temperature, the samples were read at λ = 725 nm against a blank using 1 cm quartz cuvettes. The quantitative analysis was carried out using the external standard method (gallic acid) correlating absorbance (Abs) with concentration (400–8000 mg/kg). The results were expressed in mg/kg of gallic acid.

### 2.9. Statistical Methods

We analysed survival using a Cox proportional hazard mixed model, with experimental group (i.e., Control, Control + Propolis, Control + Ethanol, *Nosema*, *Nosema* + Propolis, *Nosema* + Ethanol) as a fixed factor and cage as a random factor. The analysis was performed using the R package *coxme* [71]. We used the R packages *survival* [72] and *survminer* [73] to plot survival curves and the function *termplot* from the *stats* R package to plot hazard ratios. Statistical significance of differences in hazard ratios were evaluated using post-hoc pairwise contrasts adjusted for multiple comparisons with the Benjamini-Hochberg method to control the false discovery rate (FDR) using the R package *multcomp* [74]. We used a linear mixed model (LMM) to test for differences among treatments in log-transformed *Nosema* genome equivalents per bee and post-hoc comparison of means were again adjusted for multiple comparisons using the FDR method.

We used a generalised linear mixed effect model (GLMM) with binomial error structure to examine the effect of treatment (control vs. *Nosema*-infected) on choice of food (candy with or without propolis) in our Y maze experiment. Treatment (control vs. *Nosema*-infected), candy type (2% propolis or 5% propolis) and their interaction were used as fixed factors and the colony of origin of tested bees was used as a random factor. We also used a GLMM with binomial error structure to investigate the effects of treatment (control vs. *Nosema*-infected) on the proportion of bees feeding on the candy with or without propolis. Treatment (control vs. *Nosema*-infected), candy type (2% propolis or 5% propolis) and their interaction were used as fixed factors and cage was used as a random factor. We furthermore used a generalised linear model (GLM) to investigate the effects of treatment (control vs. *Nosema*-infected) on the proportion of propolis-candy bees consumed in their diet. All mixed effect models were performed using the *lme4* package in R [75].

All model assumptions were checked visually and were found to conform to expectations (residuals normally distributed, homogeneity of variance, linearity). All statistical analyses were conducted using R version 3.5.2 [76].

## 3. Results

### 3.1. Survival Bioassay

**Figure 1:**
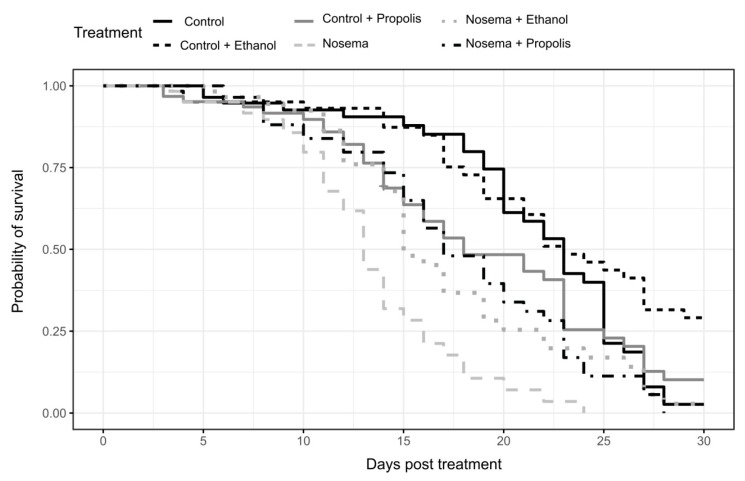
Survival of caged and experimentally treated honey bees (*Apis mellifera*): Control, Control + Ethanol, Control + Propolis, Nosema, Nosema + Ethanol and Nosema + Propolis.

**Figure 2:**
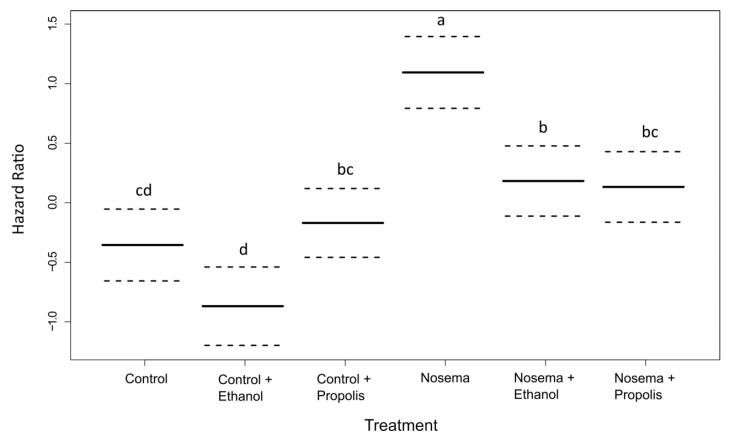
Instantaneous risk of death (hazard ratio, ± 95% CI) for adult honey bees (*Apis mellifera*) in each experimental treatment compared with the model average of 0. Different letters correspond to significant differences between treatments at *p* < 0.05 (*coxme* and post-hoc pairwise contrasts adjusted for multiple comparisons with the Benjamini-Hochberg method to control the false discovery rate).

We found a strong effect of the experimental treatment on bee survival (coxme; χ^2^ = 58.094, *p* < 0.001; Figure 1). The *Nosema* treatment caused the fastest mortality, faster than any control treatment and with 50% of bees dead within 13 days post infection (Figure 1 and Figure 2). Survival was significantly lengthened in the *Nosema* + Propolis and *Nosema* + Ethanol treatments compared to *Nosema* alone (post-hoc test adjusted for multiple comparisons with the FDR method; Z = −3.825, *p* = 0.001; Z = −3.644, *p* = 0.004; respectively Figure 1 and Figure 2, Supplementary Material Table S1), with 50% of dead individuals recorded within 17 days and 18 days post infection, respectively. The *Nosema* + Propolis and *Nosema* + Ethanol treatments were not significantly different from Control + Propolis (post-hoc test adjusted for multiple comparisons with the FDR method; *p* > 0.05; Figure 1 and Figure 2; Supplementary Material Table S1). Though ethanol seemed to extend logevity and propolis seemed to reduce longevity relative to Control, differences were not significant between Control and Control + Ethanol or between Control and Control + Propolis treatments (post-hoc test adjusted for multiple comparisons with the FDR method; *p* > 0.05; Figure 1 and Figure 2, Supplementary Material Table S1).

**Figure 3:**
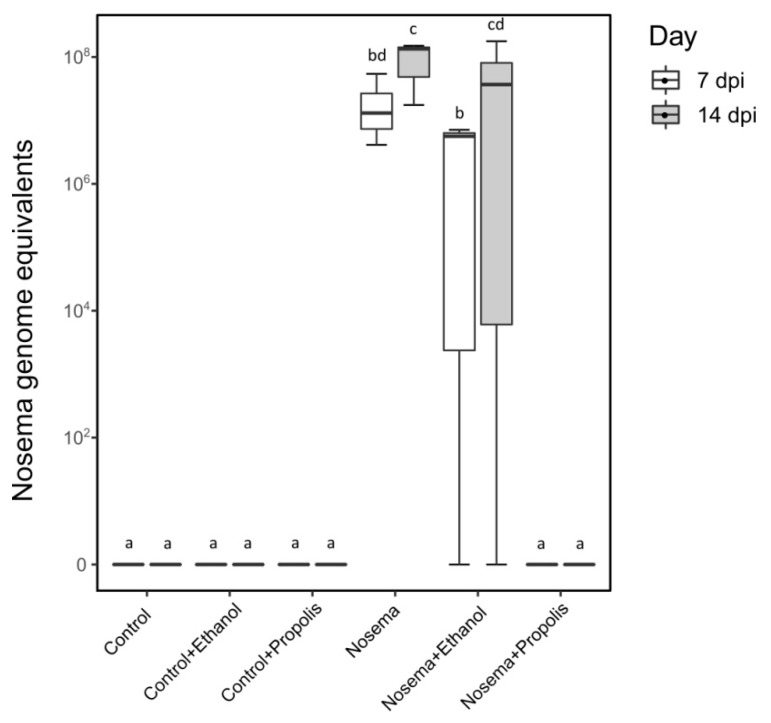
*Nosema ceranae* genome equivalents per honey bee (*Apis mellifera*) in treatments: Control, Control + Ethanol, Control + Propolis, Nosema, Nosema + Ethanol, and Nosema + Propolis at 7 and 14 days post experimental infection (dpi). Different letters correspond to significant differences between treatments at *p* < 0.05 (LMM and post-hoc pairwise contrasts adjusted for multiple comparisons with the Benjamini-Hochberg method to control the false discovery rate).

A post-hoc qRT-PCR screening of a subsample of bees collected on the 7th and 14th days post infection showed that *Nosema* infection levels differed between the treatments tested (LMM; χ^2^ = 67.997, *p* < 0.001; Figure 3). The *Nosema* and *Nosema* + Ethanol experimental groups had higher *Nosema* spore loads compared to all other experimental groups (post-hoc test adjusted for multiple comparisons with the FDR method; *p* < 0.05; Figure 3). The *Nosema* + Propolis group did not differ from the three control treatments (Control, Control + Ethanol and Control + Propolis) (post-hoc test adjusted for multiple comparisons with the FDR method; *p* > 0.05; Figure 3), indicating that this treatment group harboured little or no *N. ceranae*.

### 3.2. Food Choice Test

**Figure 4:**
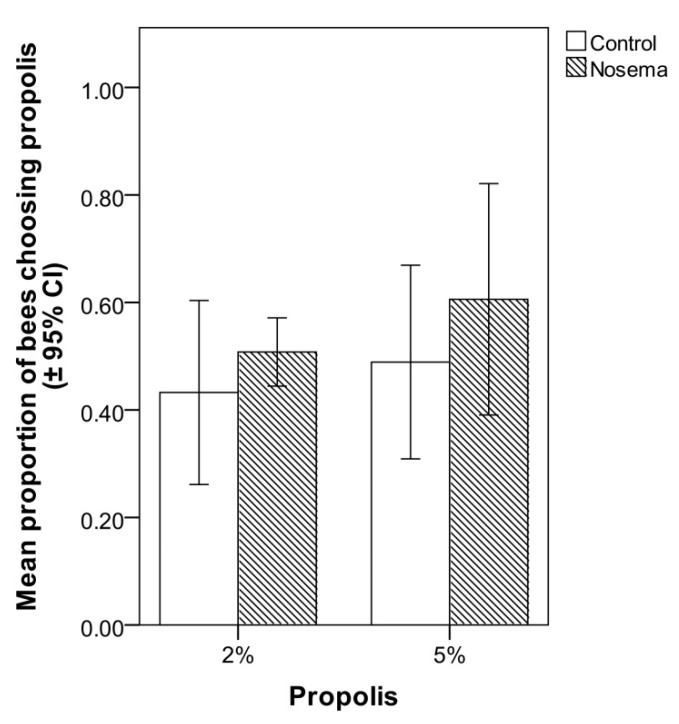
Mean proportion (± 95% CI) of honey bees (*Apis mellifera*) in *Nosema ceranae*-infected and control groups that chose candy with 2% or 5% propolis in comparison with candy without propolis in the choice test; differences between treatments were not significant (GLMM, *p* > 0.05).

In the food choice test, the addition of propolis to candy at 2% or 5% did not significantly increase its attractiveness to *Nosema*-infected or control workers (Figure 4). In fact, the proportion of bees choosing the propolis-candy rather than the candy devoid of propolis did not differ between *Nosema*-infected and control bees (GLMM; χ^2^ = 1.915, *p* = 0.166, Figure 4), candy type (with 2% or 5% propolis; GLMM; χ^2^ = 1.187, *p* = 0.275, Figure 4) or their interaction (GLMM; χ^2^ = 0.136, *p* = 0.711; Figure 4). In each bioassay, a PCR confirmed the presence of *N. ceranae* in the infected group and its absence in the control group, on both 4 and 7 days post-infection.

### 3.3. Scan Sampling and Food Consumption

**Figure 5:**
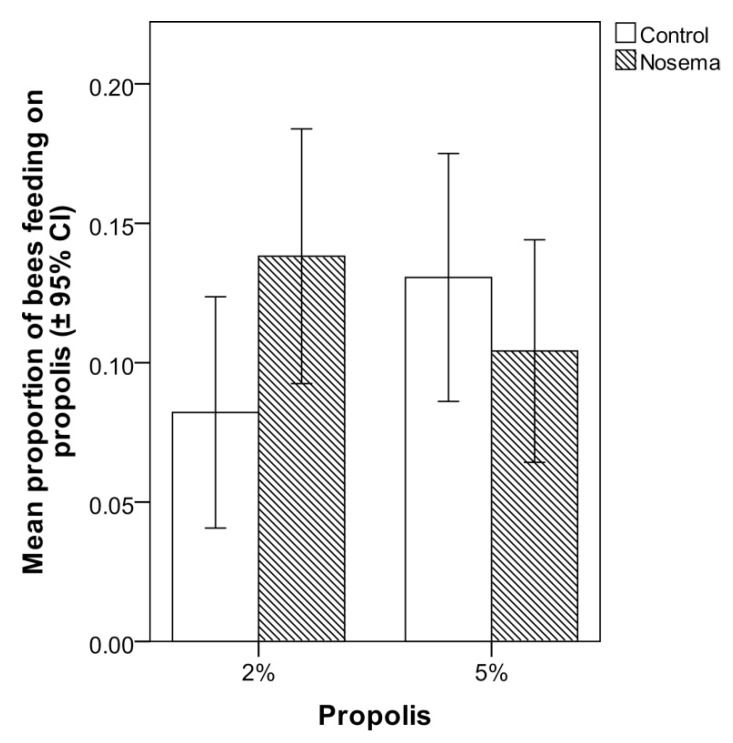
Mean proportion (± 95% CI) of honey bees (*Apis mellifera*) in *Nosema ceranae*-infected and control groups that chose to feed on the candy with 2% or 5% propolis in comparison with candy without propolis in the scan test; differences between treatments were not significant (GLMM, *p* > 0.05).

**Figure 6:**
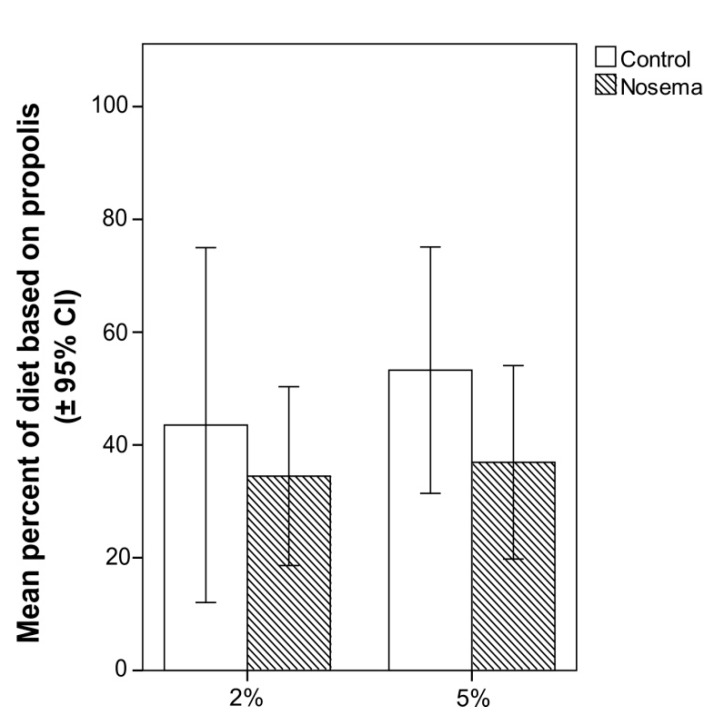
Percentage (± 95% CI) of candy with 2% or 5% propolis in the total diet consumed in *Nosema ceranae*-infected and control honey bees (*Apis mellifera*); differences between treatments were not significant (GLM, *p* > 0.05).

In the scan and food consumption tests, the addition of propolis to candy at 2% or 5% did not significantly increase the number of *Nosema*-infected or control workers that fed on it (Figure 5). In fact, the proportion of bees feeding on the propolis-candy did not differ between *Nosema*-infected and control bees (GLMM; χ^2^ = 1.188, *p* = 0.664; Figure 5), candy type (with 2% or 5% propolis; GLMM; χ^2^ = 0.070, *p* = 0.791; Figure 5) or their interaction (GLMM; χ^2^ = 0.141, *p* = 0.706; Figure 5). No significant differences were found in the proportion of propolis-candy consumed by *Nosema*-treated bees and control bees (GLM; *p* > 0.05), regardless of the concentration of propolis (2% or 5%) of the treated candy (Figure 6). In each bioassay, a PCR confirmed the presence of *N. ceranae* in the infected group and its absence in the control group, on both 4 and 7 days post infection.

### 3.4. Chemical Analysis of Propolis

HPLC analysis (Table 1) revealed the presence of almost 50 compounds belonging to the family of phenols, in particular flavones, flavonols, and simple phenols, like caffeic and ferulic acid, in the propolis extract. 

The most abundant were derivatives of quercetin, caffeic acid, ferulic acid and ellagic acid, while only small amounts of kaempherol and derivatives of cinnamic acid, rosmarinic acid and narigin were detected. The degradation pathway of larger phenols usually leads to smaller phenols [77]; in fact, it was possible to detect high levels of caffeic, ellagic and ferulic acid derivatives. However, most phenol compounds were present in low amounts. Therefore, it was very difficult to identify them unambiguously, especially because standards are not available for many of them. Nevertheless, the compounds reported here represent almost 95% of those present in the propolis extract. 

Total phenols in the propolis extract amounted to almost 120 mg/g. Differences with the total amounts obtained by HPLC analysis (Table 1) has two possible explanations, the first related to the fact that, in spectrophotometry, phenols are expressed as gallic acid whereas in HPLC they are expressed using authentic standards, and the second that phenols react differently across wavelengths. Therefore, comparison cannot be made between the two datasets.

## 4. Discussion

In this study, we evaluated the effects of propolis extracts on the lifespan and intensity of infection of *Apis mellifera* workers experimentally inoculated with *Nosema ceranae*. Our results showed that both propolis extracts and ethanol (solvent control) have a positive effect on the lifespan of *N. ceranae* infected bees. However, only propolis caused a significant reduction in *Nosema* spore load. 

Experiments similar to ours have been conducted on the red dwarf honey bee, *A. florea* [54,78], and on the Asian hive honey bee, *A. cerana* [56], using propolis produced by the stingless bee *Trigona apicalis*. In these studies, only the propolis extract and not ethanol showed positive effects on *Nosema*-infected bees, causing an increase in survival and a decrease in spore load. Similar effects of propolis on workers of *A. mellifera* infected by *Nosema* have also been reported by Arismendi et al. [57], but a different solvent was used to extract the active ingredients from propolis (methanol instead of ethanol) and the study lacked a positive control (infected bees treated with the solvent alone). In our experiments, a single administration of 2 µL of a sugar solution containing 17.5% ethanol did not cause any increase in mortality of uninfected bees nor did it change *Nosema* load in experimentally infected bees. 

Ptaszynska et al. [79] studied the impact of the prolonged administration (10 consecutive days) of ethanol on *Nosema*-infected bees, based on the fact that some beekeepers add ethanol to sucrose solution (fed in autumn to honey bees) to prevent *Nosema* infection and to cure already infested colonies. Under these conditions, they observed that the administration of sucrose syrup with 5% ethanol promoted the development of nosemosis, whereas ethanol at 10% concentration exerted severe toxic effects on uninfected bees. However, no side effects using ethanol at 2.5% concentration were observed. The acute toxicity of ethanol on honeybees was also studied by Maze et al. [80], who administered 9 μL of a 1.0 M sucrose solution containing 0, 5, 10, 25, 50, or 75% ethanol in a single dose. Maze et al. [80] observed time and dose-dependent effects of ethanol on locomotor and other behaviour (walking, stopping or walking upside down, grooming and flight behaviour), but only honey bees given the highest doses (50% and 75%) showed a significant increase in mortality. Moreover, behavioural recovery occurred between 12 and 24 h post-ingestion for low doses and at 24 to 48 h for higher doses. The total amount of ethanol supplied to each honeybee in our study (equivalent to 0.35 µL of 100% ethanol) was lower than that supplied by Maze et al. [80] at their lowest dose (equivalent to 0.45 μL of 100% ethanol). Therefore, no sub-lethal side-effects were expected in our experimental paradigm. The positive effect of ethanol on the survival of *Nosema*-infected bees observed in our study could be due to the broad-spectrum antimicrobial activity of ethanol, which includes impacts on bacteria, viruses and fungi [81]. The antifungal properties of ethanol have been recently highlighted although, at low ethanol concentrations (30%), spore germination was only partially reduced [82]. In our study, the slight, although not significant, reduction in spore loads (genome equivalents) in the *Nosema*-infected bees treated with ethanol (*Nosema* + Ethanol treatment) compared to the *Nosema* treatment might have also led to their greater survival. Because low concentrations of ethanol can enhance the activity of other biocides [81], we also hypothesize that ethanol strengthened our observed, very marked effect of propolis against *Nosema* in the *Nosema* + Propolis treatment.

The propolis used in our bioassays effectively halted the proliferation of *N. ceranae* spores in the bee gut, as previously reported by [54,55,56,57]. This is an important finding because resin chemical composition can vary with geographical area of origin and, consequently, so can its antimicrobial activity [83,84]. In general, the antimicrobial property of propolis derives from its high resin content, which is essentially associated with the content of phenolic compounds, mostly flavonoids and organic acid esters [85]. Some components isolated from Bulgarian propolis, including pinocembrin, pinobanksin-3-acetate and caffeic acid ester mixtures, are well known to be effective against the honeybee pathogens *P. larvae* and *A. apis* [86,87]. Wilson et al. [88] isolated eleven dihydro-flavonols from propolis collected in Fallon (Nevada, USA) and found that those with longer acyl groups had increased activity against *P. larvae*, whereas shorter acyl groups had increased activity against *A. apis*. To our knowledge, similar studies on *N. ceranae* are lacking. Nevertheless, several ethanolic plant extracts have shown significant anti-*Nosema* activity [57,89], probably due to their polyphenolic compound content [57,90]. Arismendi et al. [57], who chemically characterised two types of propolis that enhanced the survival of *N. ceranae*-infected bees, found many flavonol compounds (rutin, myricetin, querecetin, kaempferol and galangin) as well as phenolic acids, such as apigenin, pinocembrin and caffeic acid phenethyl ester in their propolis. Many of these compounds show broad-spectrum antimicrobial activity [91]. In contrast, chemical analysis of the propolis extract used in our study revealed the presence of mainly quercetin derivatives as well as ellagic acid, ferulic acid and caffeic acid derivatives. These differences can be explained by the environmental conditions affecting plants from which honey bees collect resins. Yet in spite of the great differences in chemical composition, propolis from different geographic locations can exhibit similar antibacterial, antifungal and antiviral activity [92]. This is to be expected, considering that honey bees are thought to use propolis to improve defense against infections. Our study demonstrates that propolis produced by honey bees in Sardinia (Italy), like that produced by honey bees from Chilean native plants [57], shows clear activity against *Nosema* spores. This suggests that the anti-*Nosema* properties of propolis in all these studies are probably due to a synergistic action of various components rather than the action of a single component.

In relation to the mechanism of action of propolis, Turcatto et al. [50] reported that propolis consumption may enhance the immune response of bees when infected with *E. coli*. Moreover, porphyrins were found to inactivate Microsporidia, preventing spore development [93]. Morphological changes in the exospore layers of treated spores indicate the direct impact of porphyrin on Microsporidia [93]. Our results, namely lack of detection of spores in infected bees treated simultaneously with propolis and *Nosema* spores, suggest a possible direct action of propolis on Microsporidia similar to that previously observed for porphyrins; propolis may have directly inhibited spore germination as opposed to reducing *N. ceranae* proliferation in host ventricular epithelial cells. However, we cannot exclude a third hypothesis, that propolis produces a thin coating on the host ventricular wall and protects bees against infection by preventing penetration of the intestinal wall by *N. ceranae*’s polar filament. Differentiating among these mechanisms of action of propolis on *N. ceranae* will require additional experimentation.

We evaluated for the first time if *Nosema*-infected bees are more attracted to a diet containing propolis, and if they consumed more of this diet in comparison to uninfected bees. We do not think that honey bees normally ingest propolis because its toxicity to them has been demonstrated [51,52], but we hypothesized that they can ingest it in moderate amounts when they are challenged by a pathogen as a form of self-medication. The lack of significant differences observed between infected and uninfected honey bees in our choice, scan and food consumption tests did not support the hypothesis that bees therapeutically use propolis in self-medication. In contrast to our findings, Simone-Finstrom and Spivak [46] and Pusceddu et al. [47] suggest propolis may be used by honey bees in self-mediation. However, these two latter studies addressed the role of propolis not against *N. ceranae* but against two other hive enemies (*A. apis* and *V. destructor*) and were conducted in an apiary rather than in the laboratory. As a consequence, both studies dealt with cases of social medication, in which the benefits and the costs derived from the use of a therapeutic substance are evaluated at the colony level rather than at the individual level [94]. In contrast, our study dealt with self-medication by bees for the benefit of the individual bee, and it is very likely that the mechanisms that regulate the response of an infected individual are different from those that regulate the response of uninfected individuals (resin foragers) to the infection of their nestmates. Propolis may therefore be used in colony-level self-medication against some pathogens and pests [46,47] but not in individual-level self-medication against *Nosema*. We note, though, that Gherman et al. [49] did observe individual self-medication by *N. ceranae*-infected honey bees, though different honeys as opposed to propolis were preferred by bees in those experiments. However, although our results did not support the idea that *Nosema*-infected honey bees consume propolis as a form of self-medication, in tests similar to those we carried out with *Nosema* in the present study, we observed that honey bee workers parasitized by *Varroa* consumed more food enriched with propolis in comparison with non-infested bees (Pusceddu et al., personal communication). Based on these findings, we think that propolis self-medication deserves further investigation. For instance, it would now be important to test diets containing propolis at different concentrations from those used in our study in protecting worker honey bees from *N. ceranae* infection.

## 5. Conclusions

The reduced proliferation of *Nosema* spores in the honey bees treated with propolis that we observed strengthens the view that it would be preferable and feasible to use natural compounds as an alternative to synthetic chemicals in the management of diseases of the honey bee and the hive, especially considering that consumers demand high-quality food products. In this context, active ingredients obtained from natural substances such as propolis seem to have the potential to control many important parasites of the hive in a safety manner. It is now necessary to conduct field studies to confirm the results observed under controlled experimental settings in order to develop a new method for the control of *Nosema* infection which would avoid the use of synthetic antibiotics in bee hives.

## Figures and Tables

**Table 1:** Total phenols (spectrophotometric) and main phenolic compounds (HPLC-DAD) in the propolis used in the bioassay.

Compound	mg/g ± RSD% ^x^
Caffeic acid ^y^	31.04 ± 5.01
Caffeic acid derivatives ^z^	113.17 ± 12.35
Paracumaric acid ^y^	7.12 ± 3.68
Ferulic acid ^y^	7.62 ± 5.25
Ferulic acid derivatives ^z^	122.70 ± 14.68
Narirutin ^y^	1.37 ± 6.51
Cinnamic acid derivatives ^z^	82.04 ± 10.78
Quercetin ^y^	10.83 ± 3.45
Quercetin derivatives ^z^	238.75 ± 6.59
Luteolin ^y^	3.00 ± 5.68
Naringin ^y^	59.01 ± 10.45
Naringin derivatives ^z^	21.99 ± 1.56
Kaempherol ^y^	34.01 ± 5.78
OH-Flavone derivatives ^z^	17.21 ± 2.56
Galangina ^y^	15.05 ± 2.48
Rosmarinic acid derivatives ^z^	44.84 ± 13.98
Ellagic acid derivatives ^z^	125.44 ± 4.65
Quinic acid ^y^	1.39 ± 8.95
Quinic acid derivatives ^z^	4.01 ± 6.01
Total Phenols *	119.77 ± 16.05

^x^ Relative Standard Deviation; ^y^ identification and quantification has been made using authentic analytical standards; ^z^ tentative identification has been made using DAD spectra similarities and quantifications were expressed as the parent compound; * expressed as gallic acid.

## Supplementary Materials

The following are available online at https://www.mdpi.com/2075-4450/11/2/124/s1, Table S1: Results of post-hoc tests adjusted for multiple comparisons with the FDR method, of differences in the hazard ratio of each treatment in the survival bioassay.
