# Supplementary material for: Propolis Consumption Reduces Nosema ceranae Infection of European Honey Bees (Apis mellifera)
Source: Insects. 2020 Feb 15;11(2):124. doi: 10.3390/insects11020124 (PMC7074184; doi:10.3390/insects11020124)
Supplement: Supplementary file 1 [file insects-11-00124-s001.pdf]

Supplementary Table S1. Results of post-hoc tests adjusted for multiple comparisons with the FDR method, for the hazard ratio of each treatment in the survival bioassay.

**Treatment comparisons**			**Estimate**	**Std. Error**	**z value**	***p* value**
Control+Ethanol	vs.	Control	-0.51323	0.25129	-2.042	0.056
Control+Propolis	vs.	Control	0.18859	0.23400	0.806	0.450
Nosema	vs.	Control	1.46227	0.24079	6.073	< 0.001 ***
Nosema+Ethanol	vs.	Control	0.54122	0.23595	2.294	0.036 *
Nosema+Propolis	vs.	Control	0.49223	0.23562	2.089	0.055
Control+Propolis	vs.	Control+Ethanol	0.70182	0.24689	2.843	0.008 **
Nosema	vs.	Control+Ethanol	1.97550	0.25856	7.640	< 0.001 ***
Nosema+Ethanol	vs.	Control+Ethanol	1.05445	0.25057	4.208	< 0.001 ***
Nosema+Propolis	vs.	Control+Ethanol	1.00547	0.25154	3.997	< 0.001 ***
Nosema	vs.	Control+Propolis	1.27368	0.23719	5.370	< 0.001 ***
Nosema+Ethanol	vs.	Control+Propolis	0.35263	0.23271	1.515	0.162
Nosema+Propolis	vs.	Control+Propolis	0.30364	0.23306	1.303	0.222
Nosema+Ethanol	vs.	Nosema	-0.92105	0.23387	-3.938	< 0.001 ***
Nosema+Propolis	vs.	Nosema	-0.97004	0.23410	0.23410	< 0.001 ***
Nosema+Propolis	vs.	Nosema+Ethanol	-0.04898	0.23360	-0.210	0.833

*** $p \leq 0.001$; ** $p \leq 0.01$; * $p \leq 0.05$
